# Macrophage targeted polymeric curcumin nanoparticles limit intracellular survival of *Mycobacterium tuberculosis* through induction of autophagy and augment anti-TB activity of isoniazid in RAW 264.7 macrophages

**DOI:** 10.3389/fimmu.2023.1233630

**Published:** 2023-07-31

**Authors:** Pramod Kumar Gupta, Priyanka Jahagirdar, Devavrat Tripathi, Padma V. Devarajan, Savita Kulkarni

**Affiliations:** ^1^ Tuberculosis Immunology and Immunoassay Development Section, Radiation Medicine Centre, Bhabha Atomic Research Centre, Mumbai, India; ^2^ Faculty of Life Science, Homi Bhabha National Institute, Mumbai, India; ^3^ Department of Pharmaceutical Sciences and Technology, Institute of Chemical Technology, Mumbai, India

**Keywords:** mycobacterium tuberculosis, autophagy, host-directed therapeutics, curcumin, multi drug resistance, phagosome lysosome fusion, apoptosis

## Abstract

Rapid emergence of antibiotic resistance in tuberculosis has left us with limited resources to treat and manage multi drug resistant (MDR) cases of tuberculosis, prompting the development of novel therapeutics. *Mycobacterium tuberculosis* (MTB) perturbs the host protective pathways for its survival, therefore host directed therapeutic (HDT) interventions offer an attractive alternative strategy. Curcumin (CMN), the principle curcuminoid from *Curcuma longa* is known to have anti-TB activity against MDR strains of MTB in macrophages. We discovered that treatment of CMN induced autophagy in uninfected and MTB infected macrophages which was evident by conversion of LC3-I to LC3-II and degradation of p62. Inhibition of autophagy by a pharmacological inhibitor 3-MA resulted in significant inhibition of intracellular killing activity of CMN, suggesting the involvement of autophagy in intracellular clearance of MTB. Moreover, annexin v-FITC/PI staining data suggested induction of apoptosis in uninfected and MTB infected macrophages post CMN treatment. This finding was further corroborated by up-regulated expression of pro-apoptotic proteins, Bax, cleaved caspase-3 and PARP and diminished expression of anti-apoptotic protein Bcl-2 as evaluated by immunoblotting. Using GFP-MTB H37Rv and Lysotracker Red staining we demonstrated co-localization of GFP-MTB H37Rv containing phagosome to lysosome after CMN treatment, indicating enhanced phagosome lysosome fusion. Due to poor bioavailability of CMN, its clinical use is limited, therefore to overcome this issue, CMN was encapsulated in Poly(lactic-co-glycolic) acid (PLGA) shell, resulting in polymeric CMN nano particles (ISCurNP). Flow cytometric evaluation suggested >99% uptake of ISCurNP after 3h of treatment. In BALB/c mice, oral dose of ISCurNP resulted in 6.7-fold increase in the bioavailability compared to free CMN. Moreover, ISCurNP treatment resulted in significant decrease in the intracellular survival of MTB H37Rv through induction of autophagy. Adjunct action of ISCurNP and CMN in combination with isoniazid (INH) revealed >99% decrease in intracellular survival of MTB in macrophage as compared to ISCurNP, CMN or INH alone. In conclusion, our findings suggest the role of ISCurNP as novel host directed formulation to combat both sensitive and MDR strains of MTB by induction of autophagy.

## 1 Introduction

Despite the availability of current drug regimen for treatment of tuberculosis (TB) for more than 50 years, TB is still one of the deadliest infectious diseases (1). Current paradigm for treatment of TB targets unique cellular processes or enzymes of *Mycobacterium tuberculosis* (MTB) associated with its survival and hence exhibit minimum toxicity to the host. However, the major disadvantage offered by this pathogen target strategy, has been the emergence of drug resistance in MTB strains (2). In the present scenario discovery and development of new drugs are outpaced by the emerging drug resistance among MTB (3). New treatment alternatives with improved efficacy and safety profile with shortened duration of therapy, are urgently needed to combat the drug resistant forms of TB, which requires a paradigm shift due to inability of current strategies to combat the drug resistance (3, 4). A significant reduction in the duration of conventional anti-TB therapy alone can have a huge impact on treatment efficacy however innovative approaches are required to bring about such changes (5). Novel approaches should exploit the recent advances made during the study of host pathogen interactions, and focus should be shifted on the drugs that do not possess inherent microbicidal activity, rather have the ability to modulate host immune responses to eliminate MTB and enhance the efficacy of current anti-TB drugs. Such approach is also known as adjunct therapy and host directed therapy (HDT) if directed towards host pathways (4, 6). A major advantage that host directed approaches offer is, miniscule possibility of development of drug resistance compared to the conventional antibiotic therapies.

Host immunity is critical to control the MTB infection, however MTB subverts the host protective immune responses to ensure its survival (7). Immune system restricts the MTB to granulomas, and thus ironically aids to the bacterial persistence in latent form within granulomas (8). Macrophages being the primary host for MTB, play central role in the TB pathogenesis, however, MTB modulates the macrophage responses in its favor. The major pathways perturbed by MTB in macrophages include phagosome lysosome fusion (9), host cell apoptosis (10, 11), autophagy (12, 13), antigen presentation (14) and macrophage activation (7).

Cellular death pathways like apoptosis and necrosis play an important role in pathogenesis of TB. Avirulent strains of MTB induce apoptosis of infected macrophages, whereas virulent strains tend to inhibit apoptosis and exhibit necrosis predominately. Apoptotic cell death in MTB infected macrophages results in intracellular killing of the pathogen and subsequent enhanced antigen presentation which eventually stimulates T cell immune response (15–18). On the other hand, necrosis of infected macrophages exacerbates the infection by release of viable MTB in microenvironment leading to spread of infection (19).

Autophagy is an important catabolic cellular pathway involved in the innate host defense against intracellular pathogens like MTB (20). Gutierrez et al. demonstrated that induction of autophagy in MTB infected macrophages caused maturation of MTB containing phagosome and subsequent fusion with lysosome which resulted into intracellular clearance of MTB (13). Virulent strains of MTB inhibit the process of phagosome lysosome fusion through several mechanisms such as exclusion of late endosome marker RAB7 GTPase or lysosomal marker LAMP-1, retention of early endosome marker RAB5 GTPase and interference in the incorporation of V-ATPase in infected macrophages (20). Autophagy is known to bypass this phagosome maturation block and it delivers MTB containing autophagosomes directly to lysosomes and thus helps in the killing of intracellular bacteria (21). Thus, any agent having the ability to induce autophagy in MTB infected macrophages may have a therapeutic potential. Antimicrobial peptide like cathelicidin LL-37 expressed in response to vitamin D3 induces autophagy in MTB infected cells (22, 23). In another study it was reported that ATP induced autophagy was involved in intracellular killing of MTB (24).

Curcumin (CMN- [diferuloylmethane or 1,7-bis (4-hydroxy-3-methoxy-phenyl) hepta-1, 6-diene-3, 5-dione)], isolated from rhizome of the herb *Curcuma longa* (turmeric) is responsible for the yellow orange color of turmeric, a spice widely used in Chinese and Indian medicine (25). Curcumin exhibits various pharmacological activities including antioxidant, antitumor, anti-inflammatory, and anti-amyloid properties (25). In previous study we reported that Curcumin

inhibits intracellular survival of sensitive as well as drug resistant strains of MTB (26) but mechanism of action was unknown. In the present study we have investigated the cellular mechanisms responsible of anti-mycobacterial activities of CMN.

Despite its wide range of therapeutic activities, the clinical potential of CMN is limited due to its hydrophobic nature, which causes poor aqueous solubility and consequent low bioavailability (27). Nanocarrier based systems have the potential to augment therapeutic effectiveness of drugs by enhancing bioavailability, possibly reducing drug resistance, and permitting cellular targeting. Such nanoparticulate systems have manifested impressive outcomes in previously reported TB studies (28–32). Poly DL, lactic–co-glycolic acid (PLGA), a biodegradable polymer, has been widely exploited for preparation of nanoparticulate formulations owing to its negligible cytotoxicity and capability to encapsulate both hydrophilic and hydrophobic drugs (33). In the present study, nanoparticles were prepared by encapsulating CMN in PLGA shell, thus providing a viable approach for its bioavailability enhancement. These nanoparticles were prepared by a highly innovative, easily scalable *in situ* method and evaluated for their potential to modulate host immune responses.

## 2 Materials and methods

### 2.1 Cell lines

Murine macrophage cell line RAW 264.7 was obtained from NCCS Pune, India and maintained in DMEM (Gibco Laboratories) supplemented with 10% FBS (Gibco Laboratories) and 1X antibiotic antimycotic solution (Sigma) at 37°C in a humidified, 5% CO_2_ atmosphere.

### 2.2 Bacterial strains and growth conditions

MDR clinical isolates of MTB, strain-1, resistant to rifampicin, ethambutol, streptomycin, pyrazinamide, kanamycin, ethionamide and strain-2, resistant to ethambutol, streptomycin, isoniazid, pyrazinamide, rifampicin, cycloserine, ethionamide, kanamycin, clarithromycin, rifabutin were obtained from patients in Tata Memorial Hospital and KEM hospital, Mumbai, India (26). Liquid cultures of MDR stains and MTB-H37Rv were grown in Middlebrook 7H9 medium supplemented with 0.05% Tween 80, 10% ADC (albumin-dextrose complex) at 37°C in a shaking incubator (180rpm) till mid log phase. Mycobacterial colonies were grown on Middlebrook 7H11 agar supplemented with 10% OADC and 0.5% glycerol during CFU assay.

Single cell suspensions of all the MTB strains growing in log phase were prepared (10^8^ bacilli/ml) as described elsewhere (34) and stored at -80°C. All procedures were carried out in a Biosafety Level III (BSL III) laboratory.

### 2.3 Animals

BALB/c mice (6–8 weeks old) mice were provided by central animal house facility, Bhabha Atomic Research Centre (BARC), Mumbai, India and all the animals were maintained in specific-pathogen-free conditions in departmental animal house facility, RMC, BARC, Mumbai, India. Mice were matched for gender and age (within 1 to 2 weeks) both, for each experiment.

### 2.4 Antibodies

Bcl-2(clone-D17C4) rabbit mAb, Bax (clone-D2E11) rabbit mAb, Caspase-3 (clone-D3R6Y) rabbit mAb, cleaved Caspase-3(Asp175, clone-5A1E) rabbit mAb, cleaved PARP (Asp214, clone-D64E10) rabbit mAb, LC3A/B (clone-D3U4C) rabbit mAb, β-Actin (clone-8H10D10) mouse mAb, and SQSTM1/p62 rabbit polyclonal Abs were purchased from Cell Signaling Technology, USA

### 2.5 Preparation of nanoparticles

Nanoparticles were prepared by previously reported *in situ* nanoprecipitation technique (35). CMN of 99% purity was obtained from Laurus Labs Limited, India as kind gift. Briefly, the preconcentrate comprising curcumin (CMN) (10 mg), PLGA (20 mg) and Soluplus (15 mg) was dissolved in DMA (0.75 mL). *In situ* curcumin nanoparticles (ISCurNP) were generated by the addition of this preconcentrate to suitable amount of filtered distilled water (filtered thru 0.22 µM filter). ISCurNP was characterized for entrapment efficiency (%EE), particle size (PS), polydispersity index and macrophage uptake prior to use.

### 2.6 Macrophage uptake

Uptake of ISCurNP by RAW 264.7 cells was monitored by flow cytometry as described (35). Briefly, cells (5 x 10^5^ cells/well) were seeded in 24-well plates treated for 3h with free CMN and ISCurNP (10 and 20 μM) or media as untreated control followed by data acquisition using flow cytometer (CyFlow Space, Sysmex). Cells were gated based on their FSC *vs* SSC, and single cells were gated based on FSC-W *vs* FSC-A. FlowJo software (Treestar) was used for data analysis.

### 2.7 Macrophage infection and CFU assay

For macrophage infection and CFU enumeration, murine macrophage cell line RAW 264.7 was used. Single cell suspensions of MTB strains were prepared as described elsewhere (34). Macrophages were co-cultured with MTB strains at MOI 5 for 4h, followed by 2 washes with pre warm media and incubated for 1h in media containing 10 µg/ml of amikacin to get rid of extracellular bacteria. Cells were again washed twice with pre warmed media and treated with CMN and/or ISCurNP at 10μM and 20μM dose for 24, 48 and 72 h. At the end of each time point, infected cells were washed twice with sterile PBS and lysed with 0.01% SDS in sterile PBS followed by CFU enumeration (34).

### 2.8 Annexin V-FITC/propidium iodide staining

Annexin V-FITC/PI staining was performed using apoptosis detection kit (BD Pharmingen) according to manufacturer’s protocol. Briefly, macrophages (5 X 10^5^ cells/well in 24-well plates) either infected with MTB strains or uninfected were treated with CMN (10 and 20 μM) or left untreated for 24h. At the end of time point, cells were harvested and stained with Annexin V-FITC and PI for 20 minutes in binding buffer (36). To prepare the single fluorochrome controls, one set of cells was treated with etoposide (1μg/ml) and stained with Annexin-V only, whereas another set of cell was incubated in water bath at 55°C for 20 minutes and mixed with live cells followed by staining with PI. Data was acquired by flow cytometer (CyFlow Space, Sysmex) and FCS express software was used for data analysis. Cells were gated based on their FSC and SSC followed by gating of single cell population based on FSC-W *vs* FSC-A. Compensation matrix was prepared and applied to all the samples at the time of data analysis.

### 2.9 Western-blot analysis

RAW cells, either infected with MTB strains or uninfected, were treated with CMN and/or ISCurNP at 10 μM and 20 μM dose for 24 and 48 h. Cells were harvested, washed thrice with 1X cold PBS and lysed in RIPA lysis buffer containing 1% protease inhibitor cocktail (Sigma) and 3% phosphatase inhibitor cocktail (Sigma). Total protein concentration in the cell lysate was estimated by Bradford reagent (Sigma). Samples containing equal amounts of protein (80μg/well) were run on 10% SDS PAGE followed by electro transfer on nitrocellulose membrane. Membranes were blocked with blocking buffer (5% w/v skimmed milk in 1X TBST) for 30 minutes followed by incubation with respective antibodies for 4 h at room temperature. Further, membranes were washed thrice with 1xTBST for 15 minutes and incubated appropriate secondary antibody HRP conjugate for 2 h and blots were developed with Enhanced Chemiluminescence kit (Roche) according to manufacturer’s protocol. Densitometric analysis was performed using ImageJ software.

### 2.10 Confocal microscopy

RAW 264.7 cells (5 x 10^5^ cells/ml) were seeded on sterile coverslips in 6-well plates and incubated at 37°C for overnight in CO_2_ incubator. Next day cells were infected with GFP-MTB H37Rv at MOI 5 and treated with CMN for 48h. Lysotracker Red (Thermo) was added to each well at concentration of 70nM for 3h prior to the end of incubation. Coverslips were washed twice with sterile 1XPBS and fixed with 4% para formaldehyde. Cover slips were mounted on glass slides using Prolong^®^ Gold Antifade (Thermo) mounting reagent containing DAPI and images were acquired on Zeiss LSM 510 Meta confocal microscope (Carl Zeiss) equipped with 63X oil immersion lens. Optical sections with Z-stack were taken to evaluate colocalization and images were deconvolved.

### 2.11 Pharmacokinetics

#### 2.11.1 Development of reverse phase HPLC method

Blood withdrawn from retro-orbital plexus of BALB/c mice was collected in 4.1% EDTA tubes. Plasma was extracted by centrifugation at 5000 rpm (10 min, 4°C). CMN from plasma was analyzed by a reverse-phase HPLC method performed on Kromasil C18 column (250 × 4.6 mm, 5 μ) under isocratic conditions on Jasco LC 2000 system (Jasco, Japan). Extraction of CMN was carried out by solvent extraction method with Hesperitin (HES) as internal standard. Briefly, 50 μL HES (500 ng/mL), 100 μL CMN (5-500 ng/mL) and 150 μL Acetonitrile were spiked to 100 μL plasma, followed by vortex mixing and centrifugation at 10000 rpm (10 min, 4°C). Mobile phase comprised 50 mM potassium phosphate buffer (pH 3): Methanol: Acetonitrile (40:30:30) at a flow rate of 1.4 mL/min. The developed method was validated for linearity, accuracy, and precision, and % CMN recovery was quantified.

#### 2.11.2 Oral pharmacokinetics study

BALB/c mice (18-20 g) aged 6-8 weeks were fasted for 12-16 h with access to water. The animals were divided into two groups: Group I- free CMN and Group II- IScurNP. Free CMN (CMN suspended in 2% HPMC) and IScurNP with CMN equivalent to 50 mg/kg body weight were administered using an oral gavage. Blood was withdrawn from retro-orbital plexus at 0.25h, 0.5h, 1h, 2h, 4h, 8h, 12h and 24h post dosing and collected in 4.1% EDTA tubes. Plasma was separated by centrifugation at 5000 rpm (10 min, 4°C) and quantified for CMN content. Plasma was spiked with 50 μL HES (500 ng/mL), 100μL methanol and 150 μL acetonitrile followed by vortexing and sonication as described in section a and CMN was quantified in the supernatant. Pharmacokinetic parameters (AUC, Tmax, Cmax) were calculated using Kinetica 5.0 software (Thermo Fischer Scientific).

### 2.12 Statistical analysis

Data obtained from independent experiments were presented as mean ± SD and analyzed by either paired Student’s t-test or one way analysis of variance (for multiple comparisons) by GraphPad Prism software (version 8). Differences were considered statistically significant at p value <0.05.

### 2.13 Ethics statement

Present study (Project no-BAEC/26/14) was approved by BARC Animal Ethics Committee (BAEC). All experiments were performed according to standard operating protocols approved and created by BAEC.

## 3 Results

### 3.1 CMN Inhibits survival of drug resistant strains of MTB by induction of autophagy

**Figure 1:**
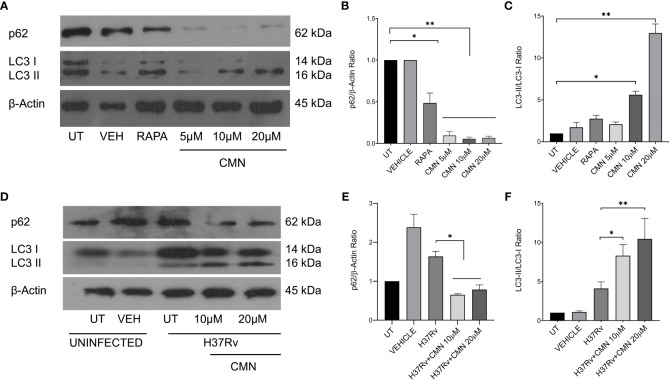
CMN treatment induces autophagy in murine macrophages. RAW264.7 cells either MTB infected or uninfected, were treated with CMN at 10μM and 20μM dose for 24h and LC3B-I and II, p62, β-actin levels were evaluated by western blotting and densitometric analysis was performed using ImageJ software. **(A)** Western blot of uninfected cells **(B)** p62/β-actin ratio **(C)** LC3-II/LC3-I ratio **(D)** Western blot of MTB infected cells **(E)** p62/β-actin ratio **(F)** LC3-II/LC3-I ratio Data representative of three independent experiments. *p<0.05, **p<0.01.

In earlier report from our lab, Gupta et al. has demonstrated that CMN inhibits intracellular survival of drug sensitive and resistant strains of MTB in murine macrophage cell line RAW 264.7 (26). Since CMN has been known to induce autophagy in various cancer cells (37–39) and autophagy is known to have anti-mycobacterial properties (13, 15, 21, 40), we investigated the effect of CMN treatment on the induction of autophagy in RAW cells. Cells were treated with CMN for 24h and conversion of LC3-I to LC3-II was monitored by immunoblotting. Conversion of LC3-I to LC3-II is one of the most important indicators of autophagy induction. Our data demonstrated the conversion of LC3-I to II at 10μM and 20μM dose suggesting autophagy induction by CMN treatment (**Figures 1A, C**). To further confirm the CMN mediated autophagic induction, degradation of p62, that is suggestive of autophagy process, was investigated by immunoblotting which demonstrated p62 degradation at 10μM and 20μM dose (**Figures 1A, B**). In next step effect of CMN treatment on autophagy induction in MTB infected RAW cells was determined. RAW macrophages infected with MTB H37Rv were treated with 10μM and 20μM dose of CMN and LC3-I to LC3-II conversion and p62 degradation was determined (**Figures 1D–F**). Our data suggest autophagy induction in MTB infected cells after CMN treatment as compared to uninfected cells. Considering the anti-mycobacterial role of autophagy, we sought to determine the involvement of CMN mediated autophagic cell death in intracellular clearance of MTB in infected macrophages by blocking autophagy by 3-Methyl adenine (3-MA) a pharmaceutical inhibitor of autophagy. RAW cells were infected with MTB H37Rv and strain-I and strain-II, treated with CMN alone and CMN in combination of 3-MA and CFU assay was performed 48 and 72h post infection.

**Figure 2:**
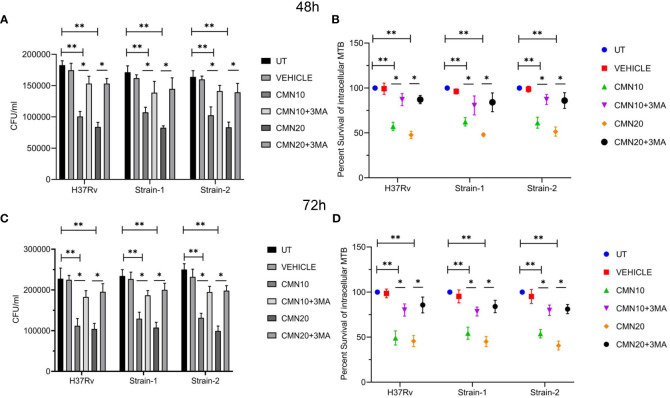
CMN inhibits intracellular survival of MTB strains by induction of autophagy. RAW 264.7 cells were infected with MTB H37Rv and two MDR clinical isolates strain1 and 2 at MOI 5, followed by treatment with CMN at dose 10μM and 20μM for 48 and 72h in presence or absence or autophagy inhibitor 3-MA. At both the time points cells were lysed and CFU assay was performed. **(A, C)** CFU/ml values of three MTB strains in presence or absence of 3-MA at 48 and 72h respectively **(B, D)** Percent intracellular survival (percentage of control). Data represented as mean ± SD, from three independent experiments. Student’s t test was used for statistical analysis (*P <0.05; **P<0.01).

A significant decrease in CFU/ml was observed in all the three strains post CMN treatment in dose and time dependent manner (**Figures 2A, C**). The percent survival of H37Rv was 57 ± 4.6 and 47.7 ± 4.1 percent (p<0.01) at 10μM and 20μM dose of CMN respectively after 48h post infection. In presence of 3-MA percent survival increased significantly to 87.1 ± 6.6 and 87.1 ± 4.6 percent (p<0.05) at 10μM and 20μM of CMN, which was comparable to untreated control (**Figure 2B**). Further, 72h post infection, CMN treatment at dose of 10μM and 20μM decreased the H37Rv percent survival to 49.1 ± 7.8 and 45.6 ± 6.1 (p<0.01) percent respectively as compared to control, however 3-MA treatment increased the percent survival in both the treatment groups to 80.1 ± 6.8 and 85.7 ± 8.8 (p<0.05, **Figure 2D**). Similar observations were made in case of both the MDR strains (Strain-1&2) wherein CMN treatment at both doses (10μM and 20μM) inhibited the percent survival at 48 and 72h time points and treatment with 3-MA caused the increase in the percent survival that was comparable to untreated controls (**Figures 2B, D**). Thus, CFU data demonstrated the involvement of CMN mediated autophagy induction in intracellular clearance of MTB strains in infected macrophages.

### 3.2 CMN induces apoptosis in MTB infected murine macrophages

**Figure 3:**
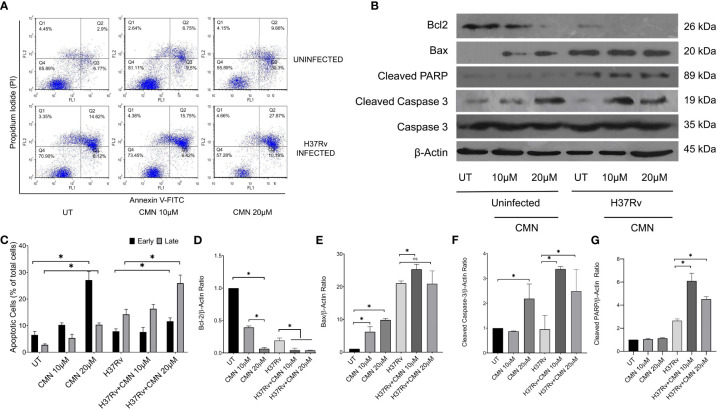
Induction of apoptosis in MTB infected murine macrophages by CMN. Uninfected and MTB infected RAW 264.7 cells were treated with CMN at dose 10μM and 20μM for 24h **(A)** CMN treated cells were stained with Annexin V-FITC/PI after 24h and data acquired by flow cytometry. Q4- Live cells, Q3- Early apoptotic cells Q2- Late Apoptotic Q1- Necrotic cells **(B)** Western blot of pro and anti-apoptotic proteins, β-actin was used as housekeeping control. **(C)** Graphical representation of early and late apoptotic cells after CMN treatment. Densitometric analysis was performed ImageJ software. **(D)** Bcl-2/β-actin ratio **(E)** Bax/β-actin ratio **(F)** Cleaved Caspase-3/β-actin ratio **(G)** Cleaved PARP/β-actin ratio. Data presented as mean ± SD from three independent experiments. (*p <0.05).

CMN is known to induce apoptotic cell death in many cancer cell lines, hence we investigated the effect of CMN treatment on apoptosis induction in RAW cells with or without MTB infection. RAW cells were infected with MTB H37Rv or left uninfected. Both infected and uninfected cells were treated with CMN at 10μM and 20μM dose for 24h. Annexin V-FITC/PI staining was performed in cells after 24h of CMN treatment and cells were subjected to flow cytometry to enumerated percentage of early apoptotic, late apoptotic, and necrotic cells. CMN treatment induced apoptosis in uninfected RAW cells in dose dependent manner with maximum percentage of early apoptotic cells at 20μM dose (P<0.05, **Figures 3A, C**). In MTB infected cells, percentage of both, early and late apoptotic cells increased significantly after CMN treatment however percentage of late apoptotic cells was much higher than early apoptotic cells (P<0.05, **Figures 3A, C**). MTB infected group exhibited enhanced cell death compared to uninfected control group without any treatment as MTB is known to induce cell death in macrophages. Next, we investigated the expression of pro and anti-apoptotic proteins in CMN treated uninfected and MTB infected cells to further confirm that cell death observed by Annexin V-FITC/PI staining is through induction of apoptosis. Cells were harvested 24h post treatment and the expression of Caspase-3, cleaved caspase-3, cleaved PARP, Bax and Bcl-2 was monitored by immunoblotting. β-Actin was used as housekeeping control. Expression of pro apoptotic proteins such as cleaved caspase-3, cleaved PARP and Bax was upregulated after CMN treatment compared to untreated cells which indicated induction of apoptosis. Further, downregulation of expression of anti-apoptotic protein Bcl-2 was observed in CMN treated RAW cells with or without MTB infection which confirmed the apoptosis induction by CMN treatment (**Figures 3B, D–G**).

The above data confirmed induction of apoptosis in MTB infected cells after CMN treatment that is known to have host protective effects for the containment and eradication of MTB in macrophages.

### 3.3 CMN induces phagosome lysosome fusion in MTB infected RAW cells

**Figure 4:**
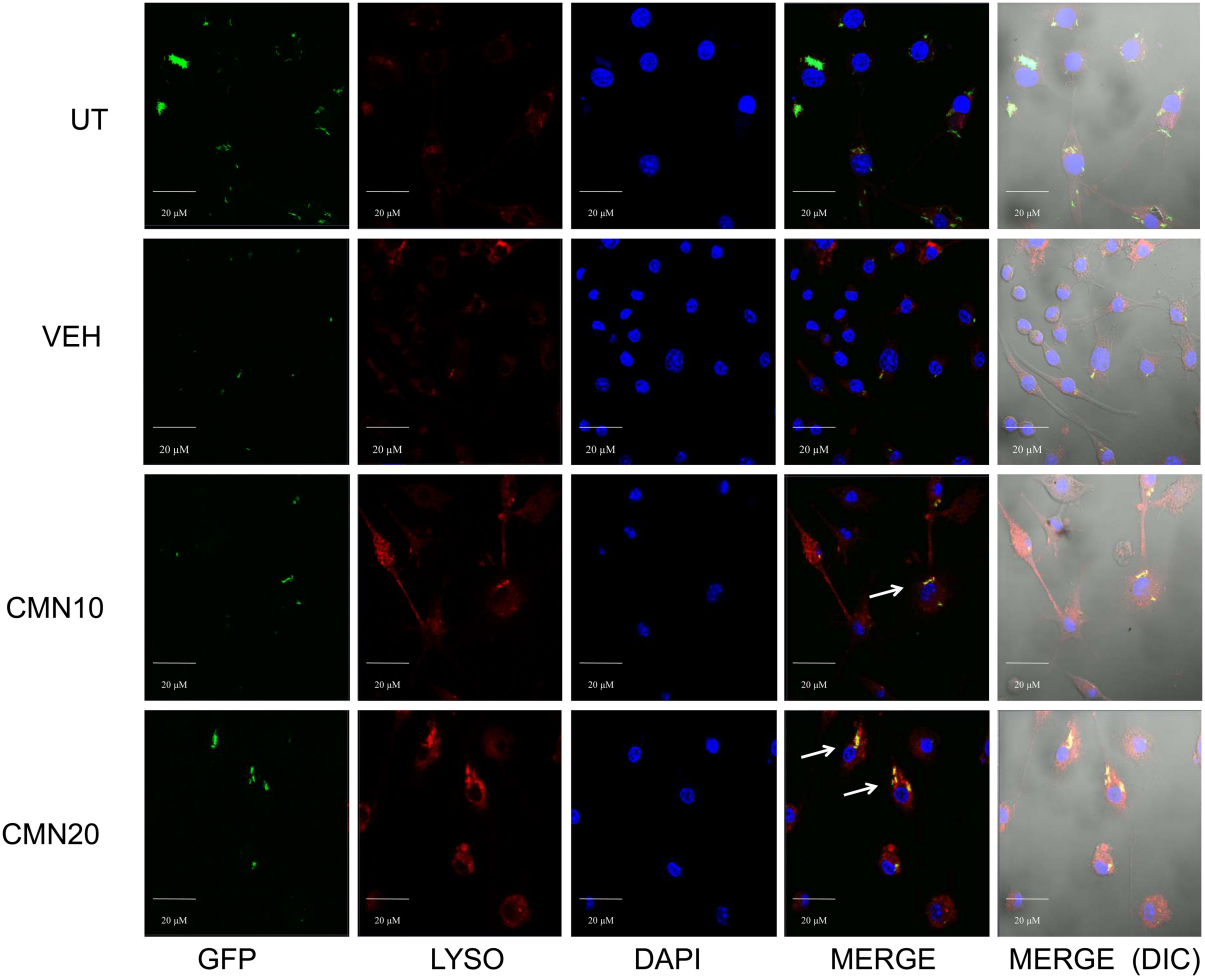
Effect of CMN treatment on phagosome lysosome fusion in MTB H37Rv infected macrophages. Murine macrophages were infected with MTB H37Rv at MOI 5 and treated with CMN at 10μM and 20μM concentration. Lysotracker Red was added to stain lysosomes and DAPI was added to stain nucleus. Scale bar - 20μm, representative images from one of the three independent experiments.

Macrophages are one of the most important constituents of innate immune system. After the phagocytosis of the pathogen, macrophage phagosomes proceed for maturation and end up into phagolysosomes after fusion with the lysosomes, leading to killing of intracellular pathogens MTB has developed several ways to ensure its intracellular survival in macrophages and perturbation of phagosome-lysosome fusion is one of the most critical means to evade the host immunity (41, 42). Apart from bacterial degradation, phagosome maturation regulates numerous immune effector functions of macrophages. Autophagy is another membrane trafficking event which can bypass the phagosome maturation blockade and deliver the mycobacterial cargo to lysosomes and helps in the elimination of intracellular MTB (21). In view of this we investigated the effect of CMN treatment on phagosome lysosome fusion in RAW cells. Cells were infected with GFP-H37Rv and lysosomes were stained with Lysotracker Red. After CMN treatment these cells were observed under confocal microscope and looked for co localization of GFP loaded phagosomal compartment (green) to Lysotracker red loaded lysosomal compartment (red). Subsequently, formation of yellow compartment indicated co localization of green and red compartments signifying the phagosome lysosome fusion. Our data demonstrated the formation of yellow compartment in both the CMN treatment groups after 48h which suggested activation of phagosome lysosome fusion. However, in untreated or vehicle control groups phagosome lysosome fusion was not observed (**Figure 4**). Thus, our data demonstrated that CMN treatment led to phagosome lysosome fusion in MTB infected macrophages and which may be attributed to CMN mediated intracellular clearance of MTB.

### 3.4 Preparation and characterization of ISCurNP

**Figure 5:**
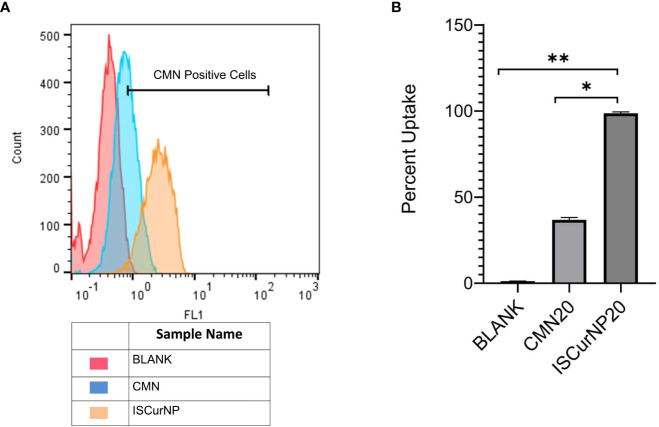
Macrophage uptake of ISCurNP by flow cytometry **(A)** Flow cytometry histogram depicting uptake of ISCurNP and CMN at 3h, **(B)** graphical representation of uptake at 3h, Data presented as mean ± SD from three independent experiments. *p<0.05, **p<0.01.

Despite the excellent host directed therapeutic potential against MTB, the therapeutic potential of CMN is limited because of its low bioavailability. To overcome this issue, CMN was converted into nano particulate form (ISCurNP), as described in our earlier report (35). Before each study, ISCurNP were freshly prepared and evaluated for particle size (PS) and percent entrapment efficiency (%EE). Typically, the PS was 212 ± 18 nm (polydispersity index <0.2) and %EE was 90 ± 2.1%. Further, flow-cytometric macrophage uptake evaluation was performed before each study, and data revealed >99% uptake of ISCurNP in RAW cells, compared with free CMN after 3h of treatment (**Figures 5A, B**).

### 3.5 Pharmacokinetics study of ISCurNP revealed enhanced bioavailability of CMN

In the next step, we determined the bioavailability of ISCurNP in BALB/c mice. Hesperitin (HES), a flavonoid, was used as an internal standard to serve as the normalizing factor for detection of CMN. HES enhanced the precision and reliability of the reverse-phase HPLC method by providing an internal check on the recovery and by reducing technical artefacts such as potential variations in injection volumes. The developed reverse-phase HPLC method for CMN in plasma revealed good resolution and separation of HES and CMN peak from peaks of other plasma proteins.

**Figure 6:**
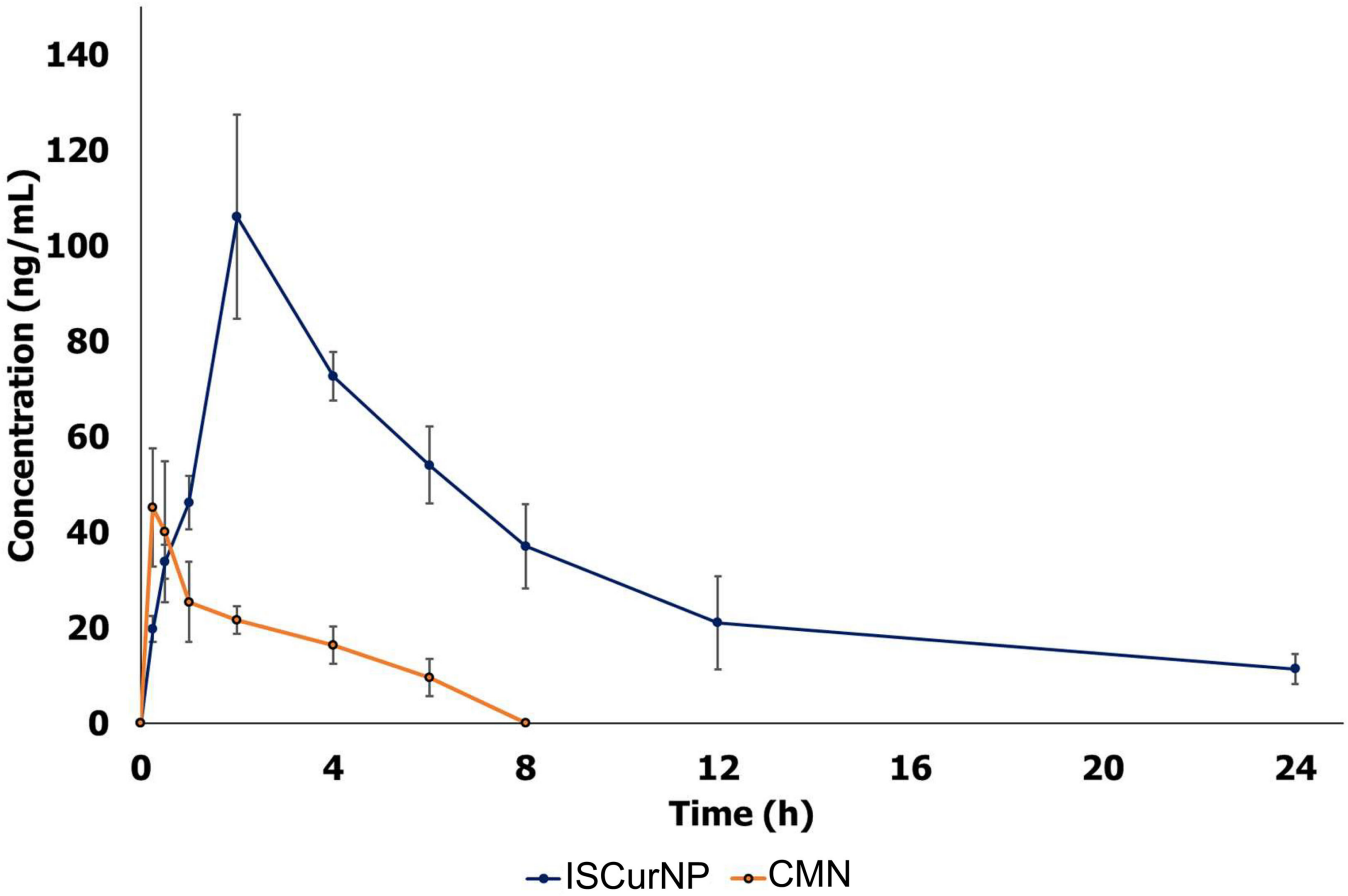
Pharmacokinetic profile of free CMN and ISCurNP after oral administration in mice. The plasma concentration versus time profiles of free CMN and ISCurNP following oral administration Data represented as mean ± SD from three independent experiments (n=6).

The plasma concentration versus time profiles of free CMN and ISCurNP following oral administration is depicted in **Figure 6**. The relevant pharmacokinetic parameters are summarized in **Table 1**. Free CMN revealed rapid absorption with a lower T_max_. An abrupt decrease in plasma CMN was observed after 30 min, which may be due to its distribution and metabolism. Free CMN was detected up to 8h after administration. In contrast, ISCurNP revealed delayed T_max_ and was detected up to 24h. This may be due to sustained release of CMN from ISCurNP (35). The C_max_ of ISCurNP increased by 2.3-fold compared with free CMN, suggesting enhanced drug absorption. Compared with free CMN, a 6.7-fold increase in the relative bioavailability of ISCurNP indicated superiority of the formulation for oral administration.

**Table 1:** Pharmacokinetics parameters after oral administration (50 mg/kg dose) of free CMN and ISCurNP in BALB/c mice.

Parameters	Treatment groups	
	Free CMN (50 mg/kg)	ISCurNP (50 mg/kg)
C_max_ (ng/mL)	45.2	106.04
T_max_ (h)	0.25	2
Half-life t_1/2_ (h)	3.66	7.014
Elimination rate constant Ke (1/h)	0.19	0.1
MRT_inf_ (h)	5.07	11.19
AUC_0–t_ (h ng/mL)	119.03	799.49
AUC_0–inf_ (h ng/mL)	169.93	914.85
Relative enhancement		6.7

AUC, area under the curve; data expressed as mean ± SD (n = 6).

### 3.6 ISCurNP treatment kills intracellular MTB by autophagy induction

**Figure 7:**
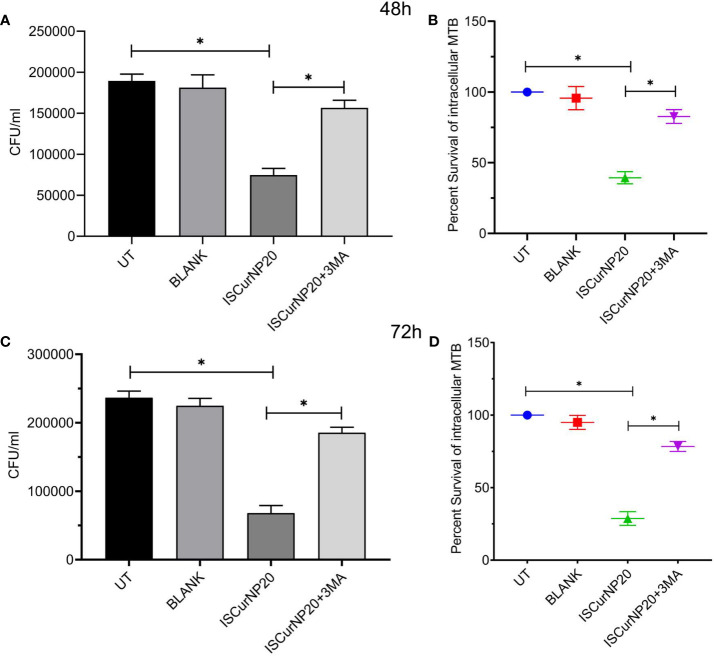
Effect of ISCurNP on intracellular clearance of MTB strains in macrophages RAW 264.7 cells were infected with MTB H37Rv at MOI 5, followed by treatment with ISCurNP at dose 20μM for 48 and 72h in presence or absence or autophagy inhibitor 3-MA and CFU was enumerated at both the time points. **(A, C)** CFU/ml values of MTB H37Rv strains in presence or absence of 3-MA at 48 and 72h respectively **(B, D)** Percent intracellular survival (percentage of control). Data represented as mean ± SD, from three independent experiments. Student’s t test was used for statistical analysis (*P <0.05).

Having evaluated the higher intracellular uptake of ISCurNP as compared to free CMN, we evaluated the anti-mycobacterial activity of ISCurNP formulation. RAW cells were infected with MTB H37Rv at MOI of 5 and treated with ISCurNP (20μM) and blank particles without CMN, for 48 and 72h or left untreated. At each time point cell lysate was plated onto MB-7H11 plates for CFU enumeration (**Figures 7A, C**). ISCurNP treated group exhibited significant decline in CFU/ml values as compared to untreated control or blank treated group. We further evaluated whether CMN mediated autophagy induction is responsible for intracellular killing of MTB in ISCurNP treated group by blocking autophagy by 3-MA. ISCurNP treated group exhibited percent survival of 39.4 ± 4.3 and 28.8 ± 4.7 (p<0.01) percent as compared to 82.8 ± 4.7 and 78.4 ± 3.4 (p<0.01) percent ISCurNP and 3-MA combination group after 48 and 72h treatment respectively (**Figures 7B, D**). Thus, it was concluded that ISCurNP inhibited intracellular survival of MTB by induction of autophagy and this data corroborated with our earlier finding using free CMN.

### 3.7 ISCurNP augmented mycobactericidal activity of isoniazid

**Figure 8:**
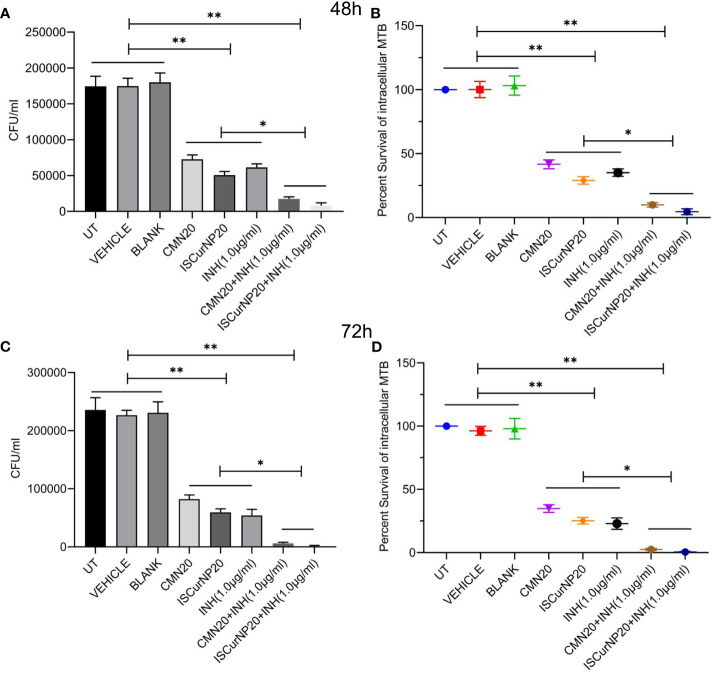
Adjunctive action of CMN and ISCurNP RAW 264.7 cells were infected with MTB H37Rv at MOI 5, followed by treatment with ISCurNP and free CMN in combination with INH or ISCurNP, CMN and INH alone, for 48 and 72h and CFU was enumerated at both the time points. **(A, C)** CFU/ml values of MTB H37Rv at 48 and 72h respectively **(B, D)** Percent intracellular survival (percentage of control). Data represented as mean ± SD, from three independent experiments. Student’s t test was used for statistical analysis (*P <0.05; **P<0.01).

In next step we investigated the adjunctive action of CMN and ISCurNP treatment to standard TB antibiotic isoniazid (INH) when treated in the combination of INH. RAW cells were infected with MTB H37Rv and treated with CMN alone, ISCurNP alone, INH alone and combination of CMN+INH and ISCurNP+INH for 48 and 72h. CFU counts of CMN+INH and ISCurNP+INH treated group decreased significantly as compared to CMN, ISCurNP and INH alone at both the time points (**Figures 8A, C**). Percent survival of MTB was 9.9 ± 1.75 and 4.85 ± 2.29 percent in CMN+INH and ISCurNP+INH treated group respectively as compared to 41.6 ± 3.5 percent in case of CMN, 29.0 ± 2.9 percent in case of ISCurNP and 35.1 ± 2.8 percent in case of INH alone after 48h of treatment (**Figure 8B**). After 72h of treatment, further decrease in the percent survival of MTB was observed and it reached to 0.56 ± 0.49 and 2.5 ± 0.85 percent in ISCurNP+INH and CMN+INH treated group respectively as compared to 34.8 ± 3.0 percent in case of CMN, 25.2 ± 2.6 percent in case of ISCurNP and 22.9 ± 4.5 percent in case of INH alone (**Figure 8D**). Thus, our data suggested that free CMN and ISCurNP both exhibited excellent adjunctive action to INH at both the time points. Adjunctive action of ISCurNP was more potent as compared to CMN as ISCurNP alone exhibited more intracellular killing of MTB as compared to CMN at both the time points which can be attributed to the higher uptake of ISCurNP as compared to CMN.

## 4 Discussion

The existing TB treatment regimen demonstrates several limitations, such as longer treatment

duration, frequent side effects of drug and low success rate. Moreover, rising incidence of drug-resistant TB creates a major bottleneck in management and eradication of TB. Exploration of novel alternative therapeutic approaches is mandatory for improving the anti-TB chemotherapy in drug resistant cases and overcome the limitations of current treatment modalities. Within this framework, an efficacious host-directed therapeutic strategy aiming to strengthen the host protective immune responses, shorten the treatment duration and diminish the treatment associated side effects, is considered vital to augment population-wide protection against the global TB burden. Adjunctive HDTs employ repurposed drugs, cytokine, vitamins, monoclonal antibodies, PRR agonists/antagonists, small molecules, and natural compounds to interfere with MTB exploited host pathways such as production of antimicrobial peptides, macrophage effector functions, T cell activation, apoptosis, autophagy and phagosome lysosome fusion (34, 43–45).

Apoptosis is one of the innate defense mechanisms subverted by the virulent MTB strains for their survival within the macrophages (46, 47) and induction of apoptosis in MTB infected macrophages is known to restrict the bacterial replication and improve T cell activation through enhanced presentation of mycobacterial antigens (48). In this scenario, targeting the apoptotic pathways to eliminate intracellular pathogens such as MTB appears interesting and candidates having potential of induce apoptosis in MTB infected cells should be investigated. In a recent study Stutz et al. reported that targeting apoptotic pathways by antagonists of inhibitors of apoptosis (IAP) proteins accelerate the *in vivo* clearance of MTB in mice. In the present study we demonstrated the induction of apoptosis in both uninfected and MTB infected RAW264.7 cells after CMN treatment. Expression of anti-apoptotic protein Bcl-2 was down regulated whereas expression of pro-apoptotic proteins Bax, cleaved PARP and cleaved Caspase 3 was up-regulated in MTB infected macrophages post CMN treatment suggesting the induction of apoptosis.

Another host defense pathway to be subverted by MTB, is phagosome lysosome fusion that helps in the degradation of mycobacterial cargo by phagosome maturation and MTB escapes the degradative phagolysosome by arresting the process of phagosome maturation (41, 42, 49, 50). Interestingly, this has garnered considerable interest in the hunt for the HDTs targeting the phagosome maturation arrest by MTB. Calcium signaling is crucial in the phagosome lysosome fusion and MTB is known to interfere with this signaling pathway to arrest the phagosome lysosome fusion. Flunarizine, a calcium modulator enhanced the intracellular killing of MTB in macrophages through the acidification and phagosome maturation *via* calmodulin‐dependent pathway making it an attractive HDT candidate (51). Using GFP tagged MTB H37Rv, we demonstrated the enhanced phagosome lysosome fusion in macrophages after CMN treatment that further suggested the HDT potential of CMN. Autophagy, an intracellular catabolic process, is crucial for maintaining the cellular homeostasis by degradation of long-lived proteins, damaged organelles, cytoplasmic portions and intracellular pathogens (52, 53). Recent evidences suggest that autophagy induction in phagocytes is an effective mechanism to control mycobacterial growth, and MTB has developed various strategies to overcome autophagy to ensure its survival (12, 54, 55). Induction of autophagy appears to have a crucial role in intracellular clearance of MTB in host cells, therefore it is worthwhile to investigate the autophagy inducing therapeutic agents as potential HDT candidates against MTB infection. In the recent past, several autophagy inducing agents such as adenosine triphosphate (ATP), vitamin D3, or, reactive oxygen species (ROS), have exhibited enhanced elimination of intracellular MTB *in vitro* (54). Till date several autophagy inducers such as tyrosine kinase inhibitors imatinib (56), ibrutinib (57) everolimus, an mTOR inhibitor (58), miR-27a antagomir (59), metformin (60), resveratrol (61), loperamide, valproic acid, carbamazepine, verapamil, and rapamycin (62) have been used to augment the eradication of MTB. Our data suggested conversion of LC3-I to LC3-II in macrophages after 24h of CMN treatment indicating autophagy induction which was further corroborated by degradation of p62 post CMN treatment. Moreover, CMN treatment induced autophagy in MTB H37Rv infected macrophages which was confirmed by conversion of LC3-I to LC3-II and p62 degradation. In next step, macrophages were infected with MTB H37Rv and two MDR strains strain-I and strain-II and 3MA was used to block the autophagy. Infected cells were treated with CMN in presence or absence of 3MA and CFU was enumerated at 48 and 72h post infection. CMN mediated autophagy was partially attributed to its anti-TB effect as a significant increase in the CFU values was observed in presence of autophagy blocker 3MA as compared to CMN alone. Interestingly, CMN treatment inhibited the intracellular survival of all the three MTB strains irrespective of their drug resistance status. Further autophagy blockage by 3MA increased the CFU values in case of all these strains, therefore our data suggested the HDT potential of CMN against sensitive as well as MDR strains of MTB.

Though CMN possesses several clinical benefits, its low bioavailability often limits its clinical use. Conversion of CMN to nano form to enhance the solubility and bioavailability is well established (63). Tousif et al. reported 5-fold higher bioavailability of CMN NPs prepared by one-step homogenization procedure compared with free CMN (64). In analogous studies, polymeric (Gantrez AN119 (65), PLGA (66–68)) or lipidic (69, 70) nano formulations of CMN revealed high bioavailability following oral administration compared with free CMN. PLGA NPs loaded with CMN increased its bioavailability by 9-fold compared with CMN administered with absorption enhancer (66). In another instance, oral administration of CMN PLGA NPs revealed a 5.6-fold higher bioavailability and improved half-life compared with the native drug (67). Similar bio enhancement of CMN encapsulated in PLGA NPs has been reported by Khalil et al. (68). We prepared PLGA based nano formulation of CMN to overcome the lower bioavailability. Our data demonstrated significant increase in the macrophage uptake of ISCurNP as compared to free CMN. Additionally, we observed a 6.7-fold increased bioavailability of ISCurNP over free CMN, suggesting superiority of the formulation for oral administration. We further performed CFU assays to evaluate the effect of ISCurNP on intracellular survival of MTB. Our CFU data demonstrated significant decrease in the intracellular survival of MTB strains after treatment with ISCurNP and use of 3MA indicated the involvement of autophagy induction in ISCurNP mediated intracellular killing of MTB strains in macrophages.

To assess the potential of CMN and ISCurNP as adjunct to the standard anti-TB therapy, we evaluated their effect on the intracellular clearance of MTB H37Rv in presence of INH. CFU data indicated significant decrease in the percent survival of MTB after treatment with CMN+INH and ISCurNP+INH combination as compared to CMN, ISCurNP or INH alone after 48h of treatment. To our surprise, at 72h time point there was more than 99 and 97 percent reduction in percent survival of MTB in ISCurNP+INH and CMN+INH combinations respectively, compared to CMN, ISCurNP or INH alone that suggested the potential of adjunctive action of CMN and ISCurNP along with INH.

In conclusion, we report here that CMN exhibit its anti-TB effect by modulating the autophagy, apoptosis, and phagosome lysosome fusion in MTB infected macrophages. ISCurNP also exhibits its anti-TB effects by modulating the autophagy in MTB infected cells irrespective of their drug resistance status. Encapsulation of CMN in PLGA shell enhances its macrophage uptake and bioavailability in mice which may help in improvement in the HDT potential of CMN. Further, its adjunctive action with INH makes ISCurNP an attractive HDT candidate, however further validation in suitable animal models will be necessary before its HDT potential can be realized.

## Data availability statement

The original contributions presented in the study are included in the article/**Supplementary Material**. Further inquiries can be directed to the corresponding authors.

## Ethics statement

The animal study was reviewed and approved by BARC Animal Ethics Committee.

## Author contributions

PG and SK conceptualized this study and designed the experiments. PJ and PD prepared and characterized the nanoparticles. PG, PJ, DT and PD conducted the bioavailability experiments and analysed the data. PG and DT performed the western blots and analysed the data. PG, PJ and SK performed the infection assays and analysed the data. PG and PJ performed the confocal experiments and analysed the data. PG, PJ and SK wrote the manuscript and every author has read edited and approved the final manuscript. All authors contributed to the article and approved the submitted version.

## Supplementary material

Supplementary Figure 1 Gating strategies of flowcytometry experiments. **(A)** Gating strategy for Annexin V-FITC/PI staining **(B)** Gating strategy of macrophage uptake.
