# Supplementary figures and images for: Macrophage targeted polymeric curcumin nanoparticles limit intracellular survival of Mycobacterium tuberculosis through induction of autophagy and augment anti-TB activity of isoniazid in RAW 264.7 macrophages
Source: Front Immunol. 2023 Jul 31;14:1233630. doi: 10.3389/fimmu.2023.1233630 (PMC10424441; doi:10.3389/fimmu.2023.1233630)

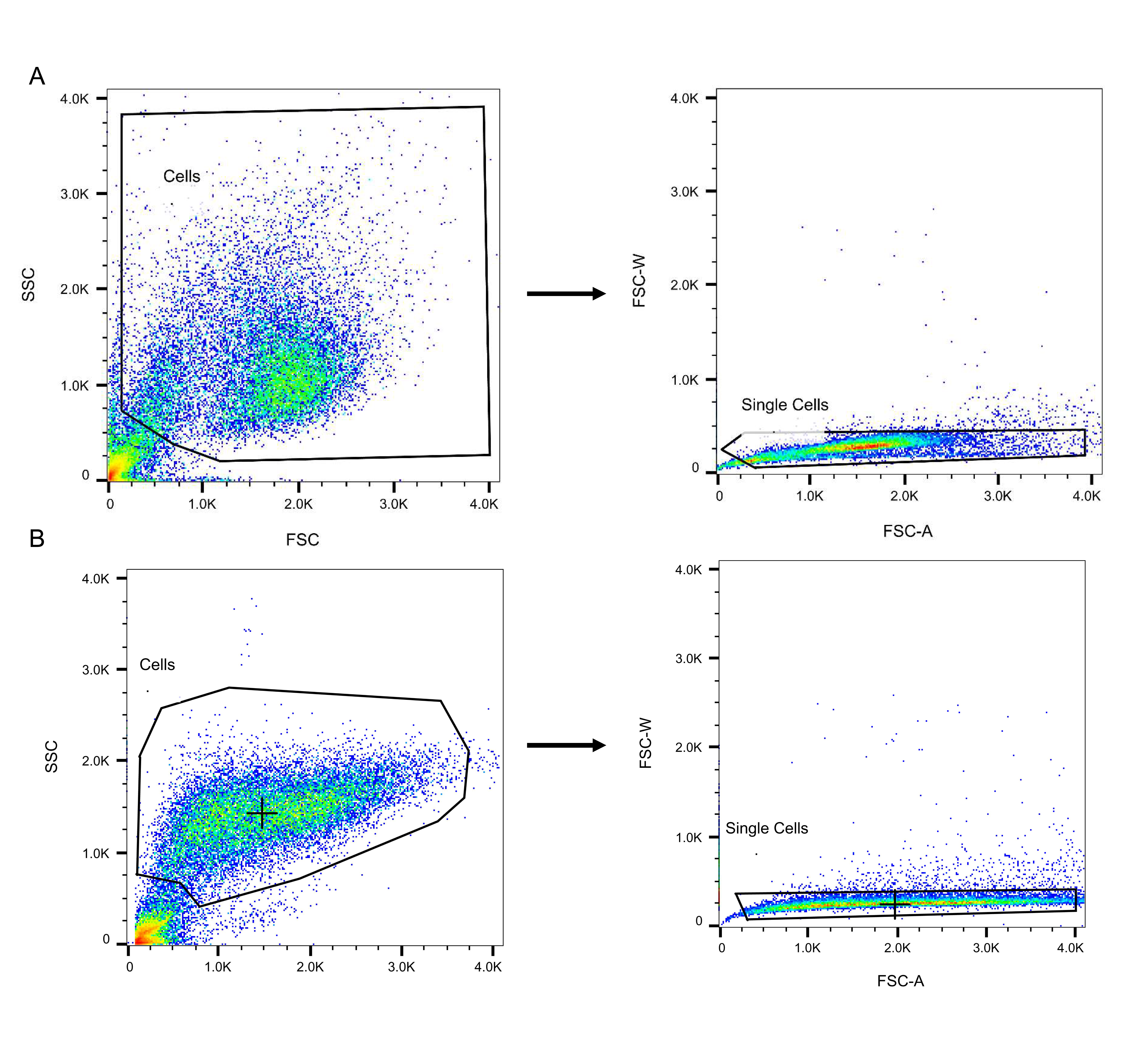

Supplement: Supplementary Figure 1 — Gating strategies of flowcytometry experiments. (A) Gating strategy for Annexin V-FITC/PI staining (B) Gating strategy of macrophage uptake. [file Image_1.tif]
